# Perspectives of patients and physicians regarding hypertensive management from an online survey for excellence: a subanalysis of the PARADOX study by physician categories

**DOI:** 10.1038/s41440-019-0365-9

**Published:** 2020-01-29

**Authors:** Takuo Yoshida, Nobuhiro Nishigaki, Shun Saita, Yukio Shimasaki, Naoyuki Hasebe

**Affiliations:** 1 Japan Medical Office, Takeda Pharmaceutical Company Limited, Tokyo, Japan; 2 Division of Cardiology, Nephrology, Respiratory and Neurology, Department of Internal Medicine, Asahikawa Medical University, Tokyo, Japan

**Keywords:** Disease management, Hypertension, Physicians, Surveys and questionnaires

## Abstract

An existing clinical problem in Japan is the high prevalence of uncontrolled hypertension despite the availability of various effective therapies. Here, we analyzed survey data to gain insight into this paradox from physicians’ perspectives, with results categorized according to specialty (i.e., with or without certification by the Japanese Society of Hypertension [JSH]), institution type, gender, and age. A web-based survey of typical educational activities for patients regarding hypertension management was conducted in Japan between October 19 and 31, 2017. Differences between physician groups were investigated per category. Survey results from 541 physicians were analyzed: 59 JSH certified (i.e., ‘specialist’) vs 482 non-JSH certified (i.e., ‘nonspecialist’) physicians; 192 general practitioners vs 349 hospital physicians; 500 males vs 41 females; and 178 younger (mean age: 40.7 years), 174 middle-aged (52.0 years) or 189 older (61.3 years) physicians. The most statistically significant differences between groups were observed in the category of physician specialty. Compared with nonspecialists, specialist physicians were more conscious of providing education on patient lifestyle modifications, more aware of patient- and physician-derived issues, and understood and followed the treatment guidelines. General practitioners cared more about the patient’s burden than did hospital physicians. Younger physicians identified the need to incorporate the patient’s perspective into their treatment. This analysis shows that the provision and perceptions of education differ between physician categories. Compared with specialist physicians, nonspecialists were less likely to provide adequate guidance on lifestyle modifications, possibly due to their uncertainty in understanding treatment guideline recommendations. Further education of nonspecialists on hypertension management may be warranted.

## Introduction

Hypertension can be managed by implementing lifestyle changes and using an array of effective and well-tolerated pharmacotherapy interventions, as recommended by guidelines from societies such as the European Society of Cardiology/European Society of Hypertension (ESC/ESH) or the American College of Cardiology/American Heart Association (ACC/AHA) [1, 2]. These recommendations are similar to the 2014 guidelines published by the Japanese Society of Hypertension (JSH), which particularly emphasize lifestyle changes as the first-line treatment for hypertension [3]. Failure to control blood pressure (BP) leads to long-term consequences of hypertension, which may include stroke, cardiovascular disease, and chronic kidney disease [1–3]. However, as the ‘hypertension paradox’ states, rates of uncontrolled BP remain high worldwide despite the growing armamentarium for hypertension treatment [4]. Similarly, in Japan, treatment success rates are not sufficiently high, remaining at ~30% in males and 40% in females in patients aged >50 years [5]. This is of concern as Japan has an aging population, and Asian patients are at particularly high risk for salt sensitivity, cardiovascular events, and stroke [6]. Thus, various approaches to overcoming this hypertension paradox are needed. Notably, the recent ESC/ESH 2018 and ACC/AHA 2017 guidelines highlight the hypertension paradox [1, 2].

To improve the treatment and control rates of hypertension and to tackle the hypertension paradox, it is important to ensure that all patients with hypertension are diagnosed and treated in a timely manner [1–3, 7]. Guidelines recommend team-based care to provide systems-level interventions involving collaboration and communication among all parties, including patients, physicians, nurses, pharmacists, and others, as necessary [1–3]. As proposed in the 2014 JSH guidelines, other factors that might increase the probability of treatment success include improving attendance at general health check-ups (incorporating hypertension screening), encouraging patients with an identified BP abnormality to attend hospital/clinic appointments, and optimizing adherence to lifestyle changes and antihypertensive medication via adequate patient education [3, 7]. In addition, from the physician’s perspective, there may be differences in their understanding and implementation of current JSH-guideline targets for BP control. A lack of specialist knowledge and experience may also contribute to therapeutic inertia, defined as the failure of the physician to intensify therapy when therapeutic goals are not reached, although multiple factors appear to underlie this phenomenon [8]. Currently, studies exploring the impact of physician expertise or specialty on hypertension management are lacking.

An online survey (PARADOX: Perspectives of patients And physicians RegArDing hypertensive management from an Online survey for eXcellence) has been undertaken in Japan to gain insight into the hypertension paradox [9]. The primary analysis, reported separately, evaluated differences between patients and physicians in their perceptions of hypertension diagnosis and management, specifically regarding the provision of education and guidance, and how patient guidance and motivation impacts adherence to lifestyle changes [10]. Here, we report results from a subanalysis of the PARADOX study, examining the differences in approaches to patient education and adherence to BP targets according to physician specialty (hypertension specialist [i.e., physicians who received certification from the JSH] vs nonspecialist), institution type (hospital-based physician vs general practitioner), gender, and age.

## Methods

### Objectives

The primary objective of this study was to investigate and analyze the gaps in approaches to hypertension management according to physician specialty (hypertension specialist [JSH certified] vs nonspecialist [non-JSH certified]), institution type (hospital physician vs general practitioner), gender (male vs female), and age (whereby respondents were split by tertiles into younger, middle, and older age groups).

### Study design

A web-based survey (PARADOX) was conducted in Japan between October 19 and 31, 2017 [10]. A subanalysis of data from the physician survey is presented here.

In brief, an online physician questionnaire, which was administered by index-i Corporation (Tokyo, Japan), consisted of 32 questions. Physicians were emailed and asked to take part in the study. Those who consented accessed the survey via an emailed hyperlink that took them to the online survey. The questionnaire used in the main survey is included in the Supplementary Documents.

### Physician survey respondents

Invited physicians were members of a research panel of Nikkei Medical Online, a members-only medical information website for healthcare professionals. As of June 1, 2018, there were ~650,000 members (of whom 157,693 were physicians [11]). It was planned that at least 500 physicians, of whom 100 had to be from cardiology departments, would be invited to complete the survey.

Initial screening questions were used to assess eligibility (Supplementary Document 1). Physicians could take part if they were aged ≥24 years, had treated ≥30 patients with hypertension in the last month and were able to prescribe treatment. Of physicians who participated in the survey, physicians were excluded from the analysis if their target systolic BP was <100 mmHg or ≥200 mmHg or if their target diastolic BP was <60 mmHg or ≥140 mmHg, based on their answers to questions 21 and 22 of the questionnaire (Supplementary Document 2).

### Statistical analysis

Differences between groups were tested by a two-sample test for equality of proportions (chi-squared test). All analyses were undertaken by index-i Corporation, in conjunction with the authors, using Microsoft Excel (Microsoft Japan Company, Limited, Tokyo, Japan).

## Results

### Demographics

Among the 565 physicians who were screened, 541 met the eligibility criteria and were included in the subanalysis (Table 1). Physician respondents were mostly male (92.4%) and nonspecialists (89.1%). Approximately two-thirds (64.5%) of the respondents were based in a hospital (hospital physicians), and one-third (35.5%) were based in a clinic (general practitioners). Demographics according to subgroup are presented in Supplementary Table 1. The mean age (range) in the younger, middle-aged and older subgroups was 40.7 (26–47), 52.0 (48–55), and 61.3 (56–95) years, respectively.

**Table 1:** Physician demographics

Physician characteristics	*N* *=* 541
Age, years	51.5 ± 9.6
Male, *n* (%)	500 (92.4)
Institution type, *n* (%)	
University or general hospital	349 (64.5)
Clinic	192 (35.5)
JSH-certified physician, *n* (%)	59 (10.9)
Specialty, *n* (%)	
Internal medicine	265 (49.0)
Cardiology	110 (20.3)
Neurology	37 (6.8)
Nephrology	33 (6.1)
Diabetes	30 (5.5)
Surgery	13 (2.4)
Gastroenterology	13 (2.4)
Respiratory medicine	10 (1.8)
Gerontology	3 (0.6)
Pediatrics	2 (0.4)
Plastic surgery	1 (0.2)
Other	24 (4.4)
Length of assessment at:	
Initial diagnosis (min)	15.6 ± 6.5
Follow-up (min)	6.6 ± 3.4
Number of patients prescribed antihypertensives in the last month (initial/follow-up visit)	167.1/141.8

Data are shown as mean ± SD or *n* (%)

*JSH* Japanese Society of Hypertension, *SD* standard deviation

### Patient education

**Fig. 1:**
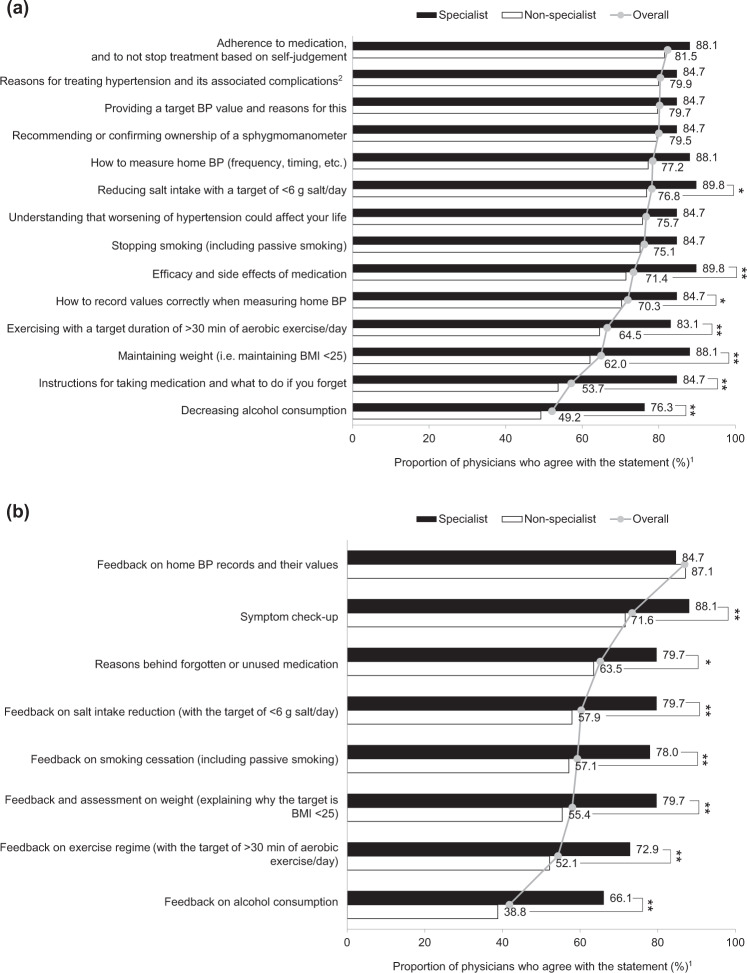
Educational topics discussed with hypertension patients at **a** initial diagnosis and **b** follow-up by specialist/nonspecialist subgroup. **a** Based on Physician Question Q3 (How thoroughly do you [the doctor] explain each of the following education and guidance factors to your patients?). **b** Based on Physician Question Q5 (How thoroughly do you confirm [or provide feedback to the patient for] each of the following symptom and lifestyle modification factors with your patients?). Respondents rated each statement from (1) very thoroughly to (5) none. Gray lines and filled circles represent the mean values of responses from all physicians (i.e., overall) for each reason. BMI body mass index, BP blood pressure. *P *<* 0.05; **P *<* 0.01. ^1^Percentage of physicians who responded ‘fully’ or ‘sufficiently’. ^2^Stroke, myocardial infarction, chronic kidney disease, etc

As shown in our previous analysis, the top three educational factors for hypertension treatment, which >80% of physicians felt that they fully or sufficiently explained at initial diagnosis, were adherence to treatment (82.3%), reasons for treating hypertension and its associated complications (80.4%), and target BP values (80.2%) [10]. Significant differences between specialist and nonspecialist physicians in patient education were observed in seven out of 14 topics (Fig. 1a). Nonspecialist physicians were less able to explain the need for lifestyle modifications, including education on daily exercise (specialist: 83.1% vs nonspecialist: 64.5%; *P* *<* 0.01), weight reduction and/or maintenance (88.1% vs 62.0%; *P* *<* 0.001), reduction of salt intake (89.8% vs 76.8%; *P* *=* 0.02), and moderation in alcohol intake (76.3% vs 49.2%; *P* *<* 0.001; all Fig. 1a). Specialist physicians were also more likely than nonspecialist physicians to provide full or sufficient information about the prescribed drug: efficacy and safety (89.8% vs 71.4%; *P* < 0.01) and actions to take if the patient missed a dose of their antihypertensive treatment (84.7% vs 53.7%; *P* < 0.001; all Fig. 1a). There was a significant difference between specialist and nonspecialist physicians in terms of the mean duration of initial consultations with patients (mean ± SD, 18.5 ± 7.2 min vs 15.3 ± 6.3 min, *P* *<* 0.01); 45.8% of specialist physicians spent ≥20 min at initial consultations compared with only 28.0% of nonspecialist physicians. When comparisons were made by grouping physicians using other criteria, a significantly higher proportion of general practitioners than hospital-based physicians fully or sufficiently explained the actions to take if antihypertensive treatments were missed (63.0 and 53.9%; *P* *=* 0.04; Supplementary Fig. 1a). Physician gender appeared to have little impact on the provision of education at initial diagnosis (Supplementary Fig. 1a). Older physicians were significantly more likely than younger physicians to fully or sufficiently explain the efficacy and safety of the prescribed drug (81.5 and 66.9% in older and younger physicians, respectively; *P* *=* 0.0014; Supplementary Fig. 1a) and what to do if an antihypertensive treatment dose is missed (66.1 and 46.6% in older and younger physicians, respectively; *P* *<* 0.001; Supplementary Fig. 1a).

Over 80% of all physicians reported that they fully or sufficiently assessed patients’ home BP diaries, and ~60% examined lifestyle changes at follow-up appointments, as demonstrated in the main analysis [10]. Similar to the results from the initial consultation, specialist physicians were significantly more likely than nonspecialists to fully or sufficiently discuss symptoms (88.1% vs 71.6%; *P* *<* 0.01), missed doses (79.7% vs 63.5%; *P* *=* 0.01), and lifestyle modifications (66.1–79.7% vs 38.8–57.9%; all *P* *<* 0.01, Fig. 1b) at follow-up. However, there were no differences observed in the duration of follow-up consultations between specialist and nonspecialist physicians (mean ± SD, 8.9 ± 5.3 min vs 6.3 ± 2.9 min). There was no difference in the proportion of physicians who confirmed BP at follow-up consultations across all categories. There were few notable differences in educational topics discussed at follow-up when the data were analyzed according to institution type (hospital or general practice), gender, or age (Supplementary Fig. 1b), although older physicians were less likely than younger physicians to discuss the reasons why a patient forgot to take their antihypertensive medication (*P* *=* 0.04; Supplementary Fig. 1b).

### Reasons for not achieving BP targets

**Fig. 2:**
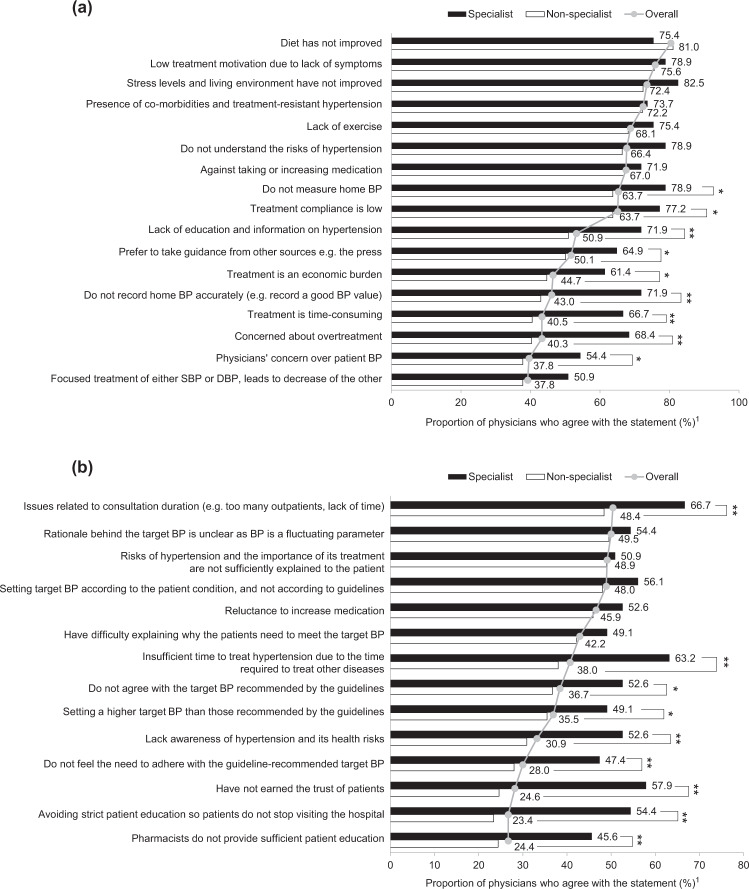
Reasons cited by specialists and nonspecialists for not achieving target blood pressure levels by subgroup: **a** patient derived and **b** physician derived. **a** Based on Physician Question Q21 (To what extent do you think each of the following patient-derived and disease-related reasons are critical factors that prevent 100% of your patients from achieving the guideline-recommended target?). **b** Based on Physician Question Q22 (To what extent do you think each of the following pharmacist- and physician-derived reasons are critical factors that prevent 100% of patients from achieving this target?). Gray lines and filled circles represent the mean values of responses from all physicians (i.e., overall) for each reason. BP blood pressure, DBP diastolic blood pressure, SBP systolic blood pressure. *P *<* 0.05; **P *<* 0.01. ^1^Percentage of physicians who responded ‘strongly agree’ or ‘agree’

As described in the PARADOX main analysis, the key patient-derived reasons for target BP not being achieved were patients’ insufficient modification of their dietary habits, low treatment motivation due to lack of symptoms, and the presence of comorbidities and treatment-resistant hypertension [10]. In terms of physician subgroups, more specialist physicians than nonspecialist physicians strongly agreed on the patient-derived reasons for not achieving the target BP level (Fig. 2a), except for improvement in diet, where a numerically higher proportion of nonspecialist physicians (81.0%) agreed than specialist physicians (75.4%). Significant differences between specialists and nonspecialists were observed for nine out of 17 of these patient-derived reasons. In particular, these differences included patients’ concern about lowering BP too much (specialist: 68.4% vs nonspecialist: 40.3%; *P* *<* 0.001), patients inaccurately recording home BP (71.9% and 43.0%; *P* *<* 0.001), patients’ concern that treatment is time-consuming (66.7% vs 40.5%; *P* *<* 0.001), and lack of patient education and information on hypertension (71.9% vs 50.9%; *P* *=* 0.003). General practitioners, compared with hospital-based physicians, were significantly more aware of patients trusting treatment advice and guidance from sources other than physicians (e.g., the press) (general practitioners: 58.2% vs hospital-based physicians: 48.1%; *P* *=* 0.03). There were no marked differences in patient-derived reasons for not achieving target BP when physicians were subcategorized by gender or age (Supplementary Fig. 2a).

Overall, ~90% of physicians identified at least one physician-derived reason for not achieving the target BP. However, more specialists agreed and identified with the physician-derived reasons than did nonspecialists, with 9 out of 14 factors being statistically significantly different (Fig. 2b). For example, in terms of specialists and nonspecialists, both physician types most frequently identified ‘not having enough time for medical examinations’ as a key factor affecting the achievement of target BP values (specialist: 66.7%; nonspecialist: 48.4%; *P* *<* 0.01). Significantly more hospital physicians than general practitioners also identified the lack of consultation time as a key factor (hospital physicians: 54.2% vs general practitioners: 43.3%; *P* *=* 0.02). However, compared with younger physicians, older physicians were less hesitant about providing strict patient education so that the patients continued to attend follow-up consultations (older physicians: 22.3%; younger physicians: 33.9%; *P* *=* 0.02; Supplementary Fig. 2b). There were no obvious differences between male and female physicians regarding physician-centric reasons for not achieving target BP.

### Perspectives on the 2014 JSH guidelines

**Fig. 3:**
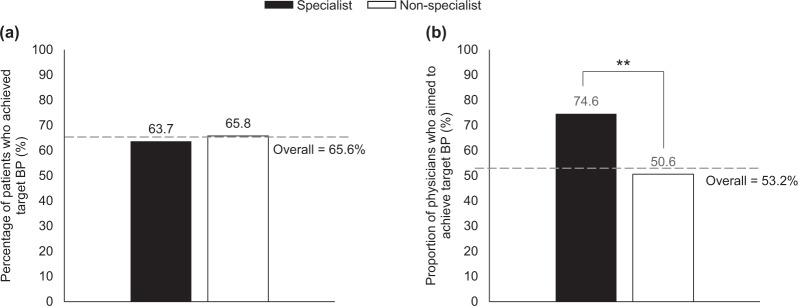
Specialist and nonspecialist physicians stating that they had **a** achieved or **b** aimed to achieve target blood pressure levels, as recommended in the 2014 JSH guidelines. The mean value represented by the dotted gray line was calculated from the response of all physicians. BP blood pressure, JSH Japanese Society of Hypertension. **P *<* 0.01

On average, 65.6% of all physicians achieved the target BP recommended by the JSH guidelines. There were no apparent differences among the subgroups (Fig. 3a and Supplementary Fig. 3a). Over half of physicians (53.2%) believed that guideline-recommended target BP should be achieved in 100% of their patients (Fig. 3b). Specialist physicians (74.6%) and hospital-based doctors (56.7%) were significantly more likely to focus on achieving target BP than were nonspecialists (50.6%; *P* *<* 0.001) and general practitioners (46.9%; *P* *=* 0.03), respectively (Fig. 3b and Supplementary Fig. 3b).

**Fig. 4:**
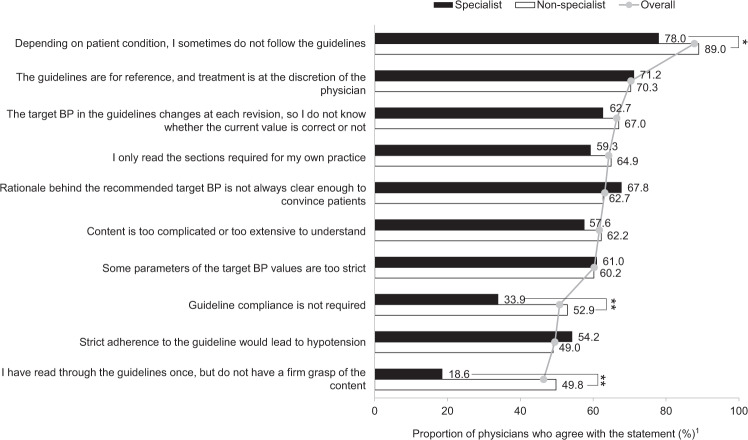
Specialist and nonspecialist opinions on the 2014 JSH guidelines: thoughts on target blood pressure levels. Based on Physician Question Q16 (Please let us know your thoughts and actions regarding the guidelines). Gray lines and filled circles represent the mean values of responses from all physicians (i.e., overall) for each reason. BP blood pressure, JSH Japanese Society of Hypertension. *P *<* 0.05; **P *<* 0.01. ^1^Percentage of physicians who responded ‘yes’

Most respondents, regardless of physician category, selected treatments based on the individual’s condition rather than adhering strictly to the guidelines (Fig. 4 and Supplementary Fig. 4). Nonspecialist physicians were significantly less likely to follow guideline recommendations (difference between groups, 11.0%; *P* *=* 0.015), significantly more likely to feel that guideline compliance is not required (difference between groups, 19.0%; *P* *<* 0.01), and significantly more likely to not have a firm understanding of the guideline content after reading (difference between groups, 31.2%; *P* *<* 0.01; all Fig. 4). Between male and female physicians, female physicians tended to be more uncertain about current target BP values, as the target values were perceived to change frequently due to guideline revisions (female: 80.5% vs male: 65.4%, *P* *<* 0.05; Supplementary Fig. 4); older physicians were more likely than younger physicians to believe that there are some occasions where the target value is too strict (older physicians: 64.0% vs younger physicians: 53.9%, *P* *<* 0.05; Supplementary Fig. 4). There were no apparent differences in physician perspectives around the 2014 JSH guidelines between hospital physicians and general practitioners (Supplementary Fig. 4).

### Future approaches to achieving the target BP

**Fig. 5:**
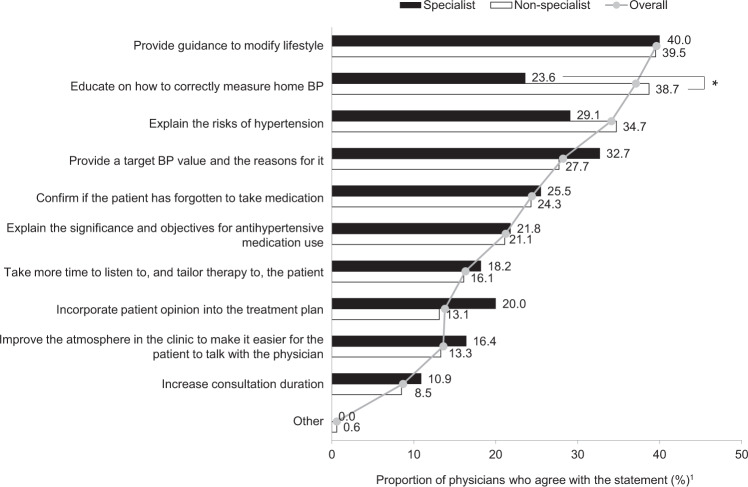
Actions that specialists and nonspecialists agreed that they may take in the future to achieve target blood pressure levels. Based on Physician Question Q23 (To resolve [these] patient-derived factors, which actions do you wish to increase efforts in more firmly in the future? Please select a maximum of three from the following options). Gray lines and filled circles represent the mean values of responses from all physicians (i.e., overall) for each reason. BP blood pressure. *P *<* 0.05. ^1^Percentage of physicians who included this factor in their top three

Overall, the top three future approaches that all physicians agreed to were improving lifestyle modification guidance (39.6%), providing education about correct home BP monitoring (37.1%), and explaining the risks of hypertension (34.1%). Few physicians felt that no future improvements were required (2.5%). More nonspecialist than specialist physicians identified future actions to achieve target BP, particularly educating about correct home BP monitoring (38.7% vs specialists: 23.6%; *P* *=* 0.03; Fig. 5). The largest difference between hospital-based physicians and general practitioners was observed around the need to create a more comfortable atmosphere to help patients feel at ease during their consultation with the physician, with more hospital-based physicians identifying improvements in the atmosphere as a step they could take toward achieving target BP (hospital-based physicians: 15.8% vs general practitioners: 9.6%, *P* *<* 0.05; Supplementary Fig. 5). Male physicians focused on lifestyle modification significantly more than female physicians did as an important future approach (40.9% vs 21.6%, *P* *=* 0.02; Supplementary Fig. 5). Older physicians were significantly more likely than younger physicians to provide future guidance on lifestyle changes (45.7% vs 32.9%, *P* *=* 0.01) and home BP monitoring to achieve target BP (44.1% vs 32.4%, *P* *=* 0.03; both Supplementary Fig. 5). Interestingly, older physicians were less likely than younger physicians to incorporate patient opinions into the treatment plan (9.7% vs 20.6%, *P* *<* 0.01).

## Discussion

Overall, this analysis of the PARADOX study outlines potential differences in the delivery of patient education, in perceptions surrounding the reasons for not achieving target BP levels, and in the acknowledgement of, and opinions on, target BP according to physician specialty, institution type, gender, and age.

At the initial consultation, the majority of physicians (regardless of subgroup) acknowledged and provided education to patients on education items included in the 2014 JSH-guideline treatment strategy, such as the importance of adherence to treatment, reasons for treating hypertension and its associated complications, and educating patients about target BP [10]. Ensuring that patients received appropriate education around the benefits of good BP control has been shown to improve adherence to lifestyle and drug interventions [12–16]. In the current survey, specialists provided significantly more guidance to patients around lifestyle management factors than did nonspecialists in four key self-management areas: daily exercise (specialist: 83.1% vs nonspecialist: 64.5%, *P* *<* 0.01), weight reduction and maintenance (88.1% vs 62.0%, *P* *<* 0.001), decreased salt intake (89.8% vs 76.8%, *P* *=* 0.02), and moderation of alcohol intake (76.3% vs 49.2%, *P* *<* 0.001). These differences may lead to a subsequent treatment success rate, as suggested by previous studies, which demonstrated that the specialty of the treating physician may partly influence educational initiatives on adherence and treatment success [12, 17–19].

The trend was similar at follow-up consultations, whereby significantly more specialists than nonspecialists provided patient education at follow-up in all factors examined (all *P* *=* 0.01 or *P* *<* 0.01; except feedback on home BP records and their values). Furthermore, nonspecialists placed less emphasis than specialists did on the practical aspects of hypertension management, including how to cope with a missed dose and the implementation of lifestyle changes. It may be difficult for nonspecialists to provide patient education at the same level as specialists, as demonstrated by the significant and large difference in the well-known lifestyle modification factor, decreasing salt intake (*P* *<* 0.01). The average salt intake in the Japanese population has gradually decreased in recent years (to 10.4 g salt per day in a national survey in 2011 from 25 g per day in the Tohoku region in the 1950s) [3], yet the current intake is still far greater than the <6 g salt per day JSH recommendation [3, 20]. Smoking cessation, a strategy that is actively implemented in Japan along with decreasing salt intake, is recognized by both specialists and nonspecialists as an important area of education. We speculate that in Japan, smoking cessation is more widely promoted and acknowledged than changes to other lifestyle habits, and BP control by patients may improve if the reduction in salt intake is recognized as being as important as smoking cessation.

The differences described above in patient education provided at initial consultations may be due to the significantly longer initial consultation period of specialist physicians compared with nonspecialist physicians. Of note, a larger proportion (45.8%) of specialist physicians spent ≥20 min with patients during the initial consultation, compared with only 28.0% of nonspecialist physicians. This length of time may increase the opportunity to discuss important educational topics with the patient during the initial consultation, thereby increasing patient awareness. Frequent and prolonged feedback at follow-up consultations are a key intervention tool used to alter patient behavior [1–3]. There were fewer differences in other physician subgroups (institution type, gender, and age) than those observed between specialists and nonspecialists concerning educational provision. Therefore, to improve patient education practices in the future, there is a potential need to support nonspecialists in providing education to their patients regarding lifestyle changes. We hypothesize that nonspecialists may receive less information than specialists about hypertension treatment. Patient education improvements for patients treated by nonspecialists could be achieved by involving the patient’s immediate family for support or through targeted educational initiatives mobilizing a team of medical professionals (e.g., pharmacists, nutritionists, and hypertension and/or cardiovascular disease therapists) or smartphone applications [1, 2, 21].

A number of patient- and physician-derived barriers to achieving target BP levels and the differences in these factors between categories were identified in our study. Specialist physicians were more broadly aware of issues leading to missed target BP levels than were nonspecialist physicians. Consequently, in their daily practice, specialists may be consciously searching for patient-derived issues that may lead to a low success rate compared with nonspecialists. Furthermore, general practitioners appeared to be significantly more conscious of patient-centric factors that were indirectly related to hypertension (e.g., stress, economic burden, and inaccurate recording of home BP; all *P* *<* 0.05) than were hospital-based physicians. It may be effective to consider patient-centric factors to improve patients’ lifestyles, given the significant association between socioeconomic factors and urinary sodium-to-potassium ratios in residents in Japan [22].

In addition to the barriers identified for achieving target BP levels, the overwhelming outcome of the survey was that the overall rate of achievement of JSH-recommended target BP level was only 66%. This is higher than previously published results from the NIPPON DATA study, in which ~30 and 40% of males and females, respectively, achieved a BP of <140/90 mmHg [5]. The differences between the two studies may be due to the timing of data collection (2010 for NIPPON DATA and 2018 for our study) and data sources (patient surveys were conducted for NIPPON DATA, while our study surveyed physicians); however, we consider that even 66% is still not sufficiently high. Furthermore, our study showed that physicians did not strictly adhere to guideline-recommended target BP values (Fig. 4). Based on the guidelines, physicians should aim for the lower target BP for most patients if aggressive treatment is tolerated [1–3]. We hypothesize that physicians may need to aim for a target BP that is lower than guideline-recommended targets to reach target BP and overcome inertia. Moreover, alternative therapeutic options may be required for patients who do not respond to antihypertensive treatments.

No differences between physician subgroups were observed when the rate of target BP achievement was examined (Fig. 3a). However, there were differences in the comprehension of the JSH guidelines according to physician specialty and institution-type subgroups (Fig. 4; Supplementary Fig. 4). As expected, significantly more specialist vs nonspecialist physicians felt that guidelines should be adhered to (*P* *=* 0.01) and understood the guidelines after reading them thoroughly (*P* *<* 0.001); however, ~60% of all physicians, regardless of specialty, found that the content of the guidelines was complicated. In addition, both specialists and nonspecialists felt that the recommended target BP level was too strict, suggesting the need for improved guidance on this point in the future; in particular, significantly more nonspecialists responded that they do not follow the guideline recommendation (depending on the patient’s condition). The results from our study indicate that nonspecialist physicians may not fully appreciate and understand the background that led to the setting of the target BP. Therefore, real-world analyses and responses to guideline recommendations have been mixed [23–26]. In Japan, for example, recommendations for hypertension management were changed in 2009; however, an analysis showed there was little change in the prescription patterns of antihypertensive drugs [26, 27]. Similar trends were observed in the UK and Canada, where separate analyses concluded that physicians required more education on achieving the target BP recommended by their respective guidelines [24, 25].

Physicians in all subgroups felt that guidance around lifestyle modification and home BP measurement and the reiteration of the risks of hypertension were key future strategies that they would like to incorporate into their practices to ensure better hypertension management. Although similar proportions of specialists and nonspecialists fully or sufficiently educated patients on home BP measurements (Fig. 1a), more nonspecialists felt that improvements could be made in the future (Fig. 5). We hypothesize that this finding may have arisen because nonspecialists are less confident in the guidance that they provide for patients and, as discussed previously, this could be improved with further education. In addition, hospital-based physicians identified the need to improve the surroundings during patient consultations compared with general practitioners (*P* *=* 0.05). We hypothesize that general practitioners, as a function of their role, will have a better understanding of, and rapport with, their patients than will a specialist physician who practices in hospitals and who may only consult a patient after a referral received from a general practitioner. Although hospital-based physicians have access to advanced medical treatment, they may feel that patients’ needs are not satisfied by only providing advanced care; we inferred from our results that hospital-based physicians may have begun to note that responding to patient needs, e.g., providing a comfortable atmosphere at consultation, are required for patients to ultimately achieve good BP control. The physician’s gender did not affect the level of initial guidance provided regarding lifestyle modifications (Fig. 1); however, one difference observed when physicians were asked about future strategies was that male physicians more frequently identified strengthening lifestyle modification messages than did female physicians (*P* *=* 0.02). This may be because female physicians may be more patient-centered in their communication than male physicians are, resulting in their ability to provide guidance according to each patient’s individuality and situation [28–30].

While we observed the greatest differences in responses between specialists and nonspecialists, whether physician-based factors (such as specialty, institution type, or gender) substantially influence hypertension care is unclear from the literature. A systematic review of 49 studies showed that for patients with a single discrete medical condition, there was no clear consensus regarding whether specialist or generalist care gave any advantages over the other [31]. Another study showed that in patients with type 2 diabetes, those treated by female physicians achieved their target blood pressure more often than did patients treated by male physicians [32].

As the current study was an online survey based on the participating physicians’ self-assessments, the answers provided may lack objectivity and may have some inaccuracies. The survey was also limited to physicians who are able to use and have access to the internet. The actual BP data of the patients were not collected in this survey, and the rates of achievement of target BP may be inaccurate. In addition, physician responses were not matched to patient outcomes. If matched responses were available, they may have provided greater insight into the potential impact of the different treatment practices observed.

In conclusion, comparison analysis between subgroups showed that the most pronounced difference was observed when specialists were compared against nonspecialists regarding the delivery of and perceptions around patient education. Compared with nonspecialists, specialists were more likely to provide detailed patient education and were more conscious of guidance on lifestyle modifications. It may thus be necessary to consider physician categories when examining factors that may contribute to the hypertension paradox. In the future, further education of nonspecialists in treatment guideline recommendations, especially regarding lifestyle changes and the background behind the recommended target BPs, may improve treatment success in Japan.

## Supplementary information

Supplementary Digital Material

Supplementary Figure 1

Supplementary Figure 2

Supplementary Figure 3

Supplementary Figure 4

Supplementary Figure 5

Supplementary Table 1

Supplementary Document 1

Supplementary Document 2
