# Supplementary material for: Perspectives of patients and physicians regarding hypertensive management from an online survey for excellence: a subanalysis of the PARADOX study by physician categories
Source: Hypertens Res. 2020 Jan 29;43(5):431–41. doi: 10.1038/s41440-019-0365-9 (PMC8075984; doi:10.1038/s41440-019-0365-9)
Supplement: Supplementary file 1 — Supplementary Digital Material [file 41440_2019_365_MOESM1_ESM.docx]

## Supplementary Digital Material

**Supplementary Fig. 1** Educational topics discussed with hypertension patients at **a** initial diagnosis and **b** follow-up, by subgroup.

**Supplementary Fig. 2** Reasons cited by physician subgroup for not achieving target blood pressure levels, by subgroup: **a** patient derived and **b** physician derived.

**Supplementary Fig. 3** Specialists and non-specialist physicians stating that they had **a** achieved or **b** aimed to achieve target blood pressure levels, as recommended in the 2014 JSH guidelines.

**Supplementary Fig. 4** Physician opinions on the 2014 JSH guidelines: thoughts on target blood pressure levels, by subgroup.

**Supplementary Fig. 5** Actions that physicians agreed they may take in the future to achieve target blood pressure levels, by subgroup.

**Supplementary Table 1** Physician demographics, by subgroup.

**Supplementary Document 1** Physician survey questionnaire.

**Supplementary Document 2** Physician screening questionnaire.
