# Supplementary figures and images for: Perspectives of patients and physicians regarding hypertensive management from an online survey for excellence: a subanalysis of the PARADOX study by physician categories
Source: Hypertens Res. 2020 Jan 29;43(5):431–41. doi: 10.1038/s41440-019-0365-9 (PMC8075984; doi:10.1038/s41440-019-0365-9)

Supplementary Figure 1

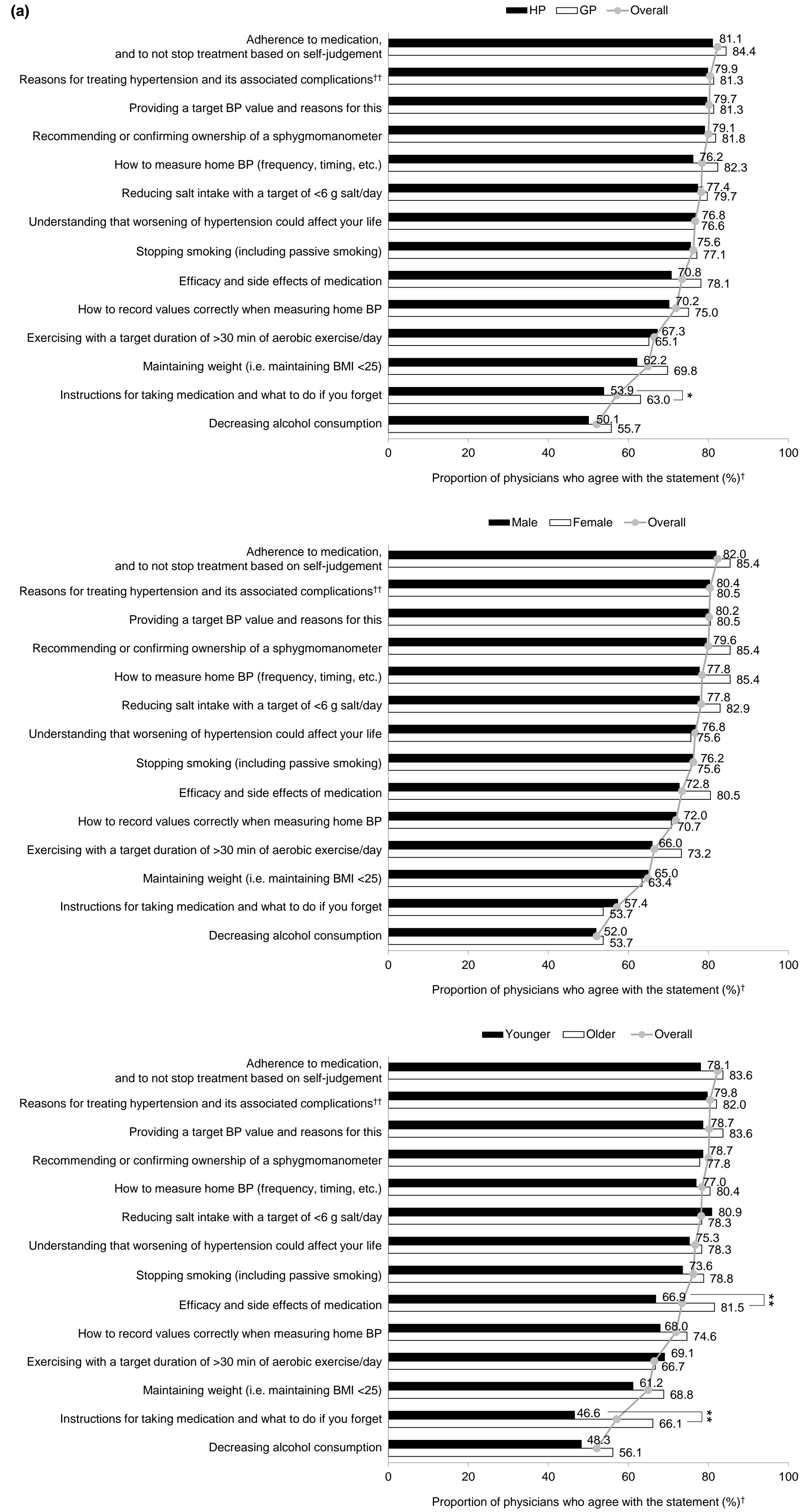

(b)

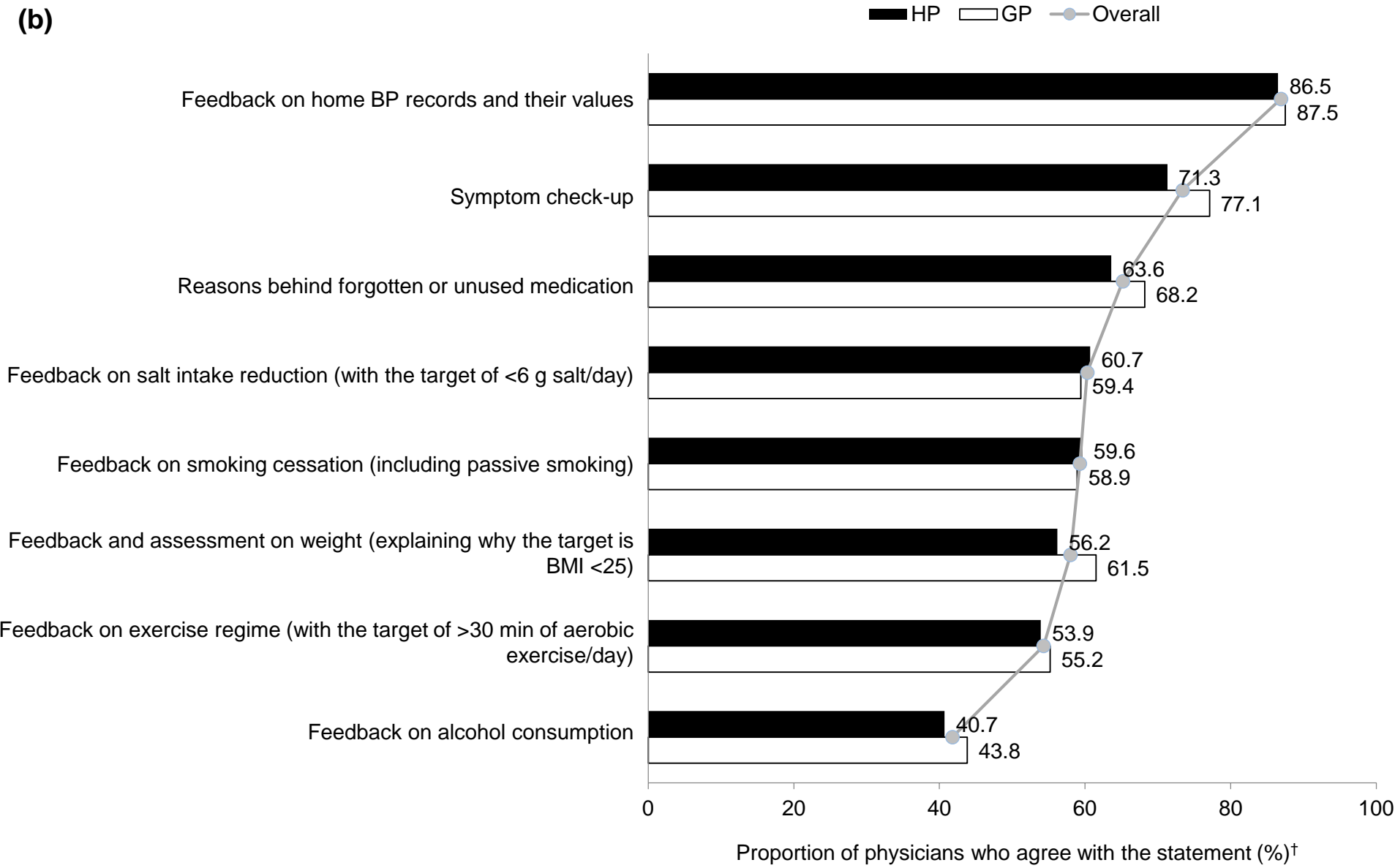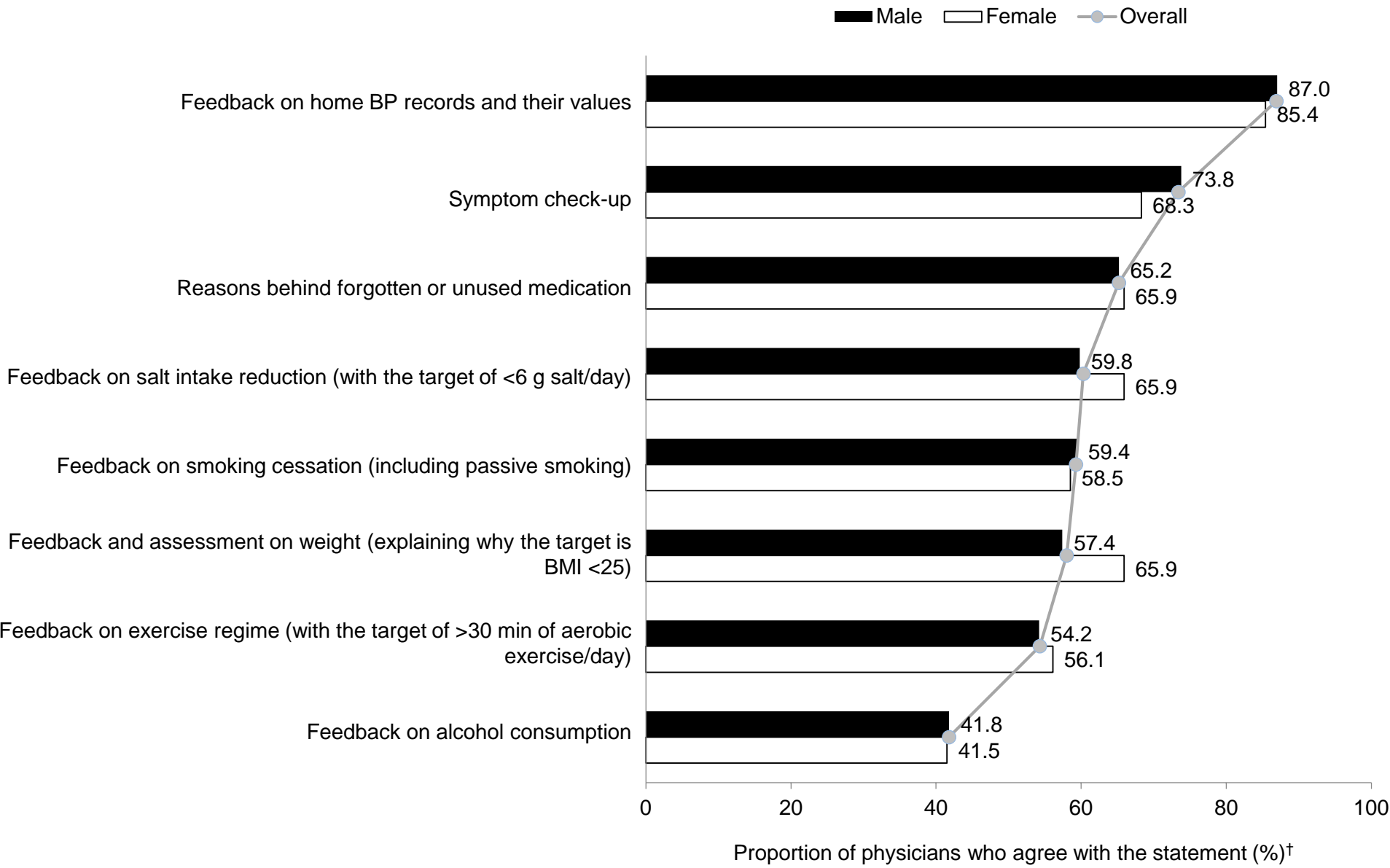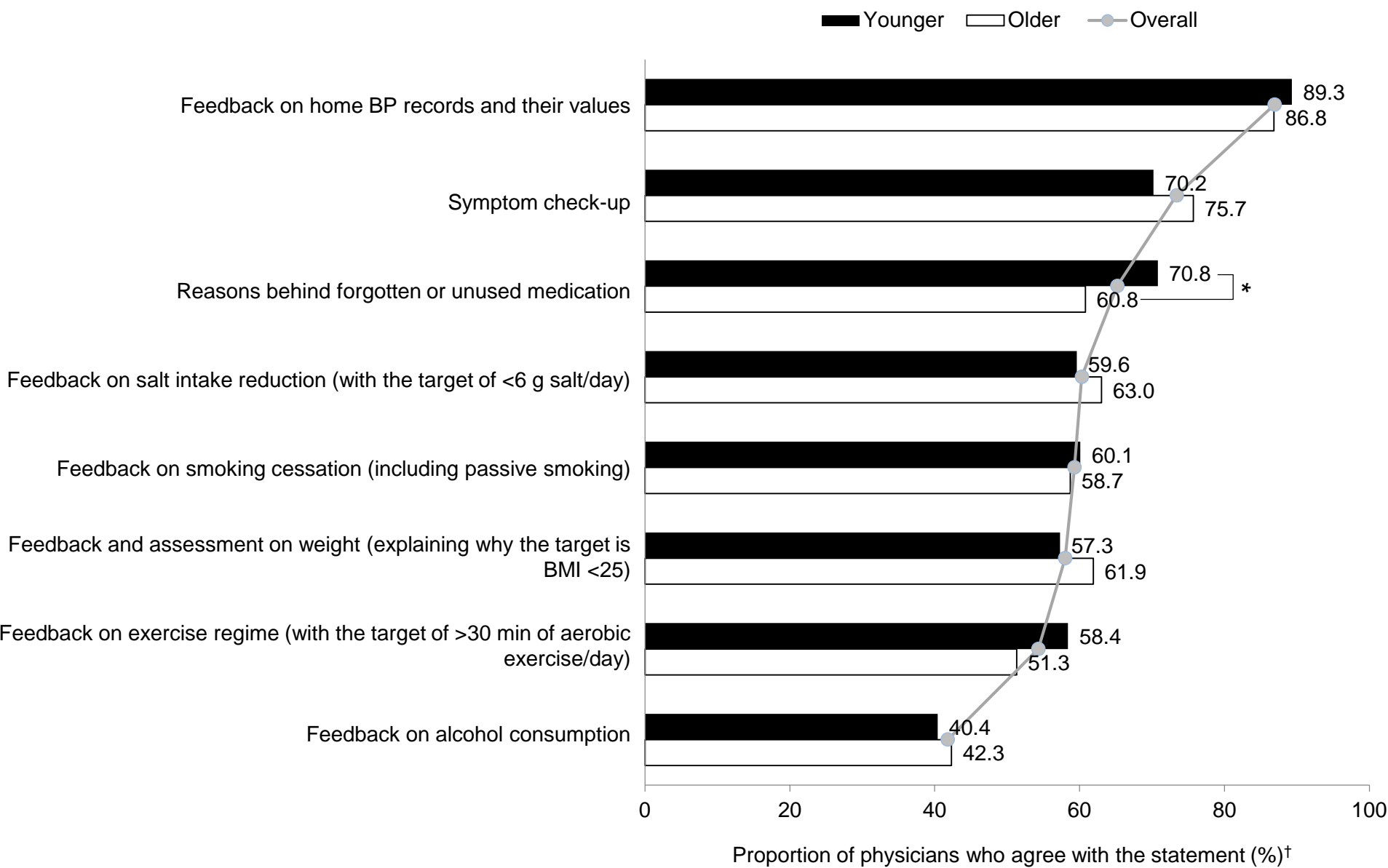

Supplement: Supplementary file 2 — Supplementary Figure 1 [file 41440_2019_365_MOESM2_ESM.pdf]

Supplementary Figure 2

(a)

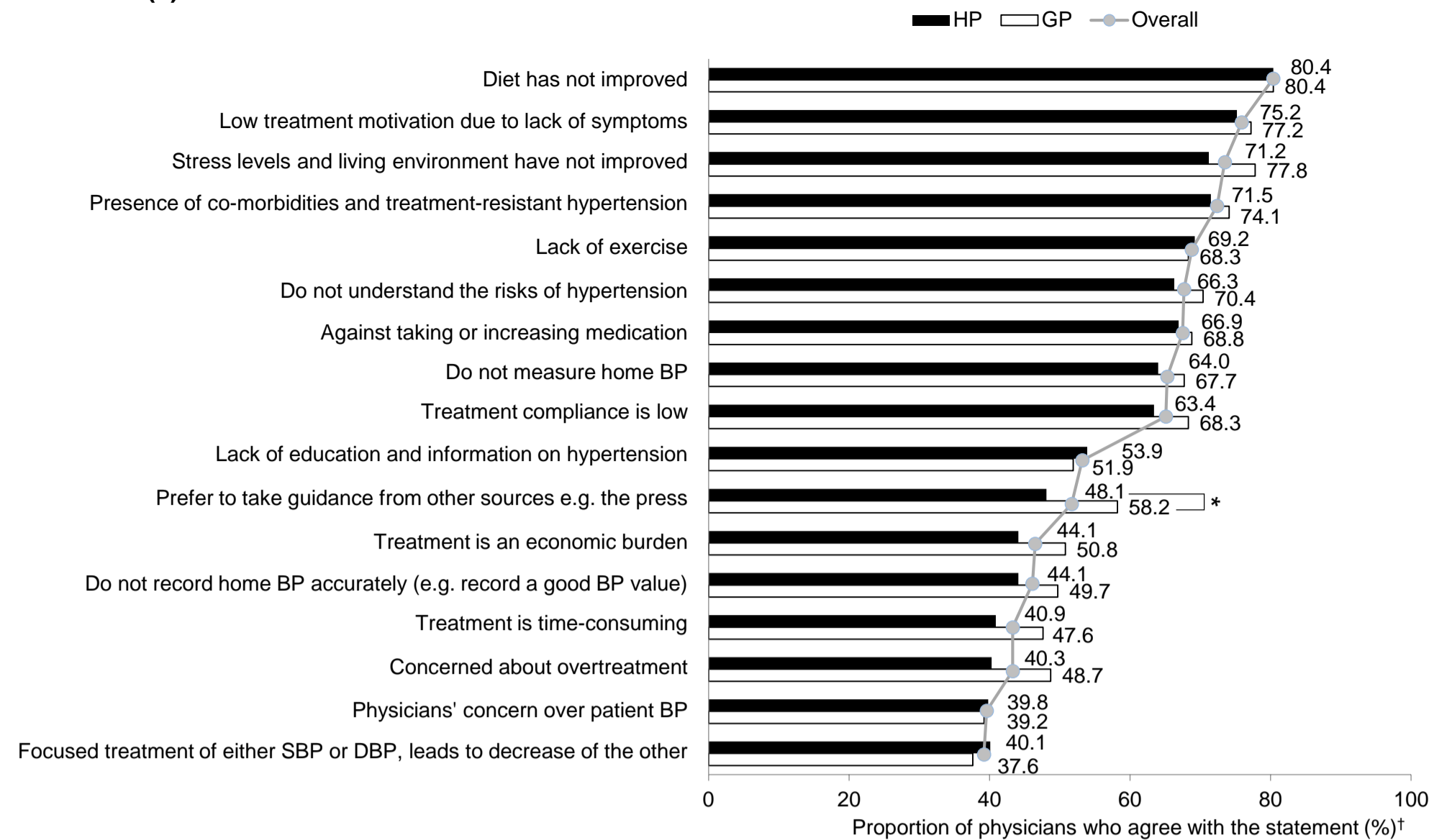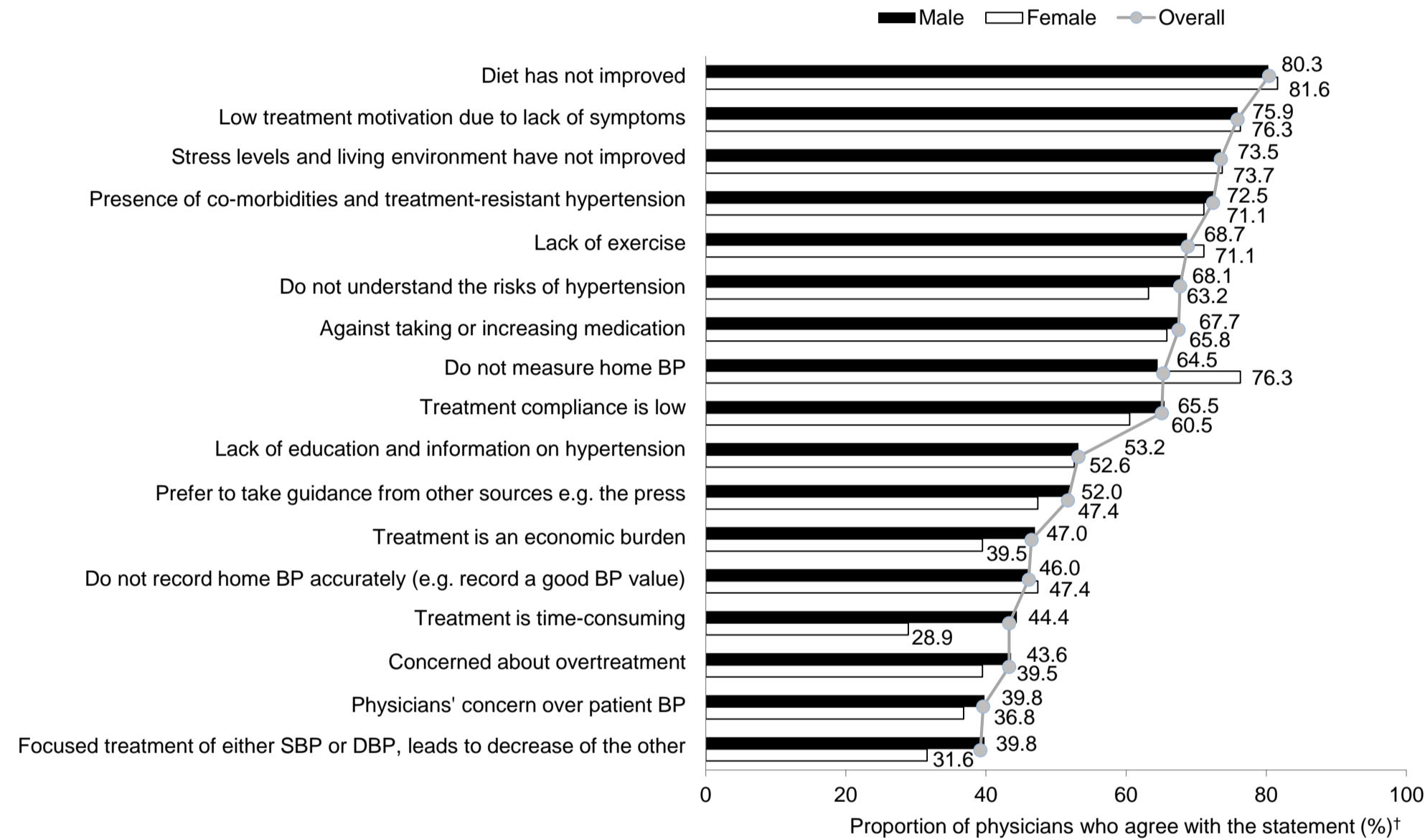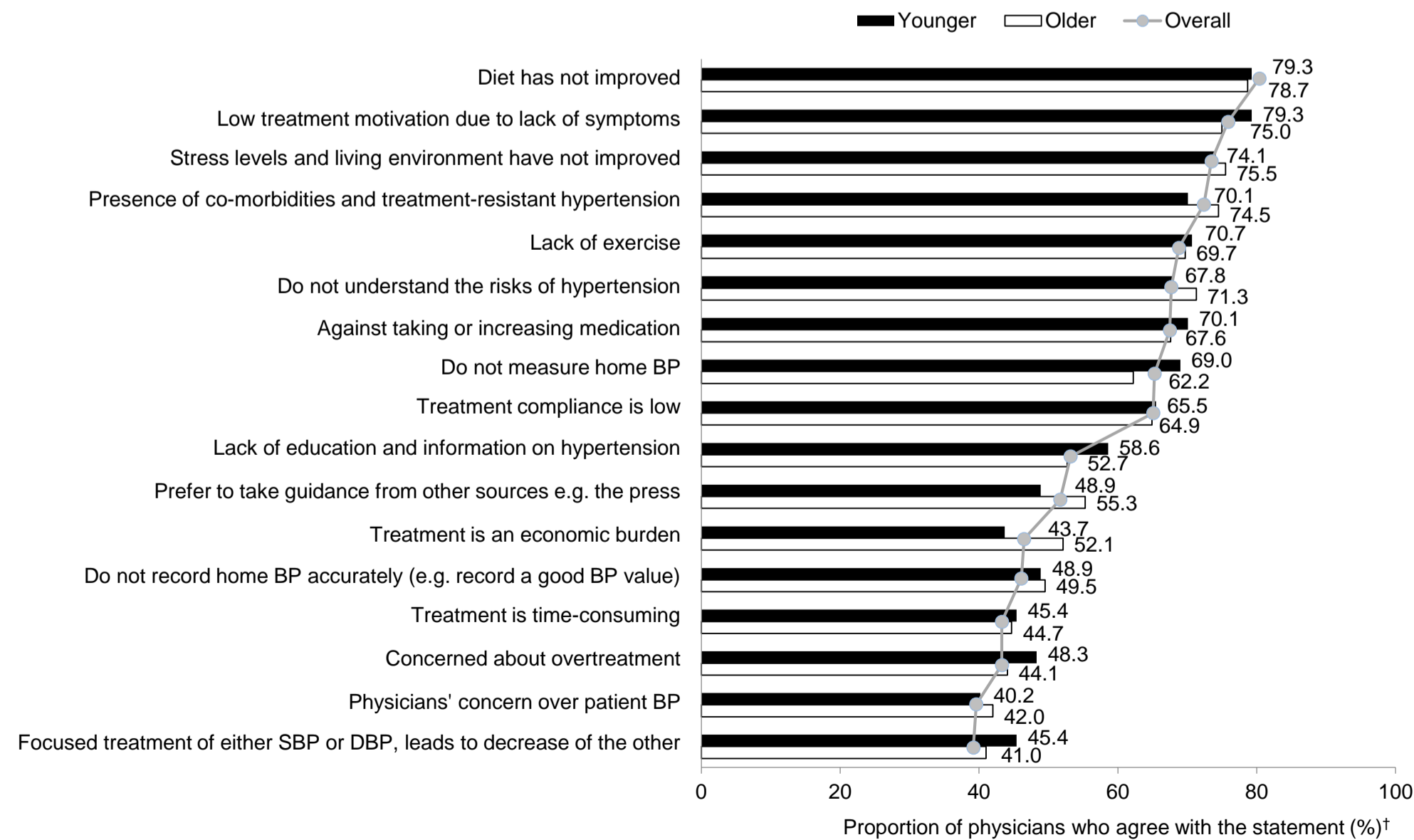

(b)

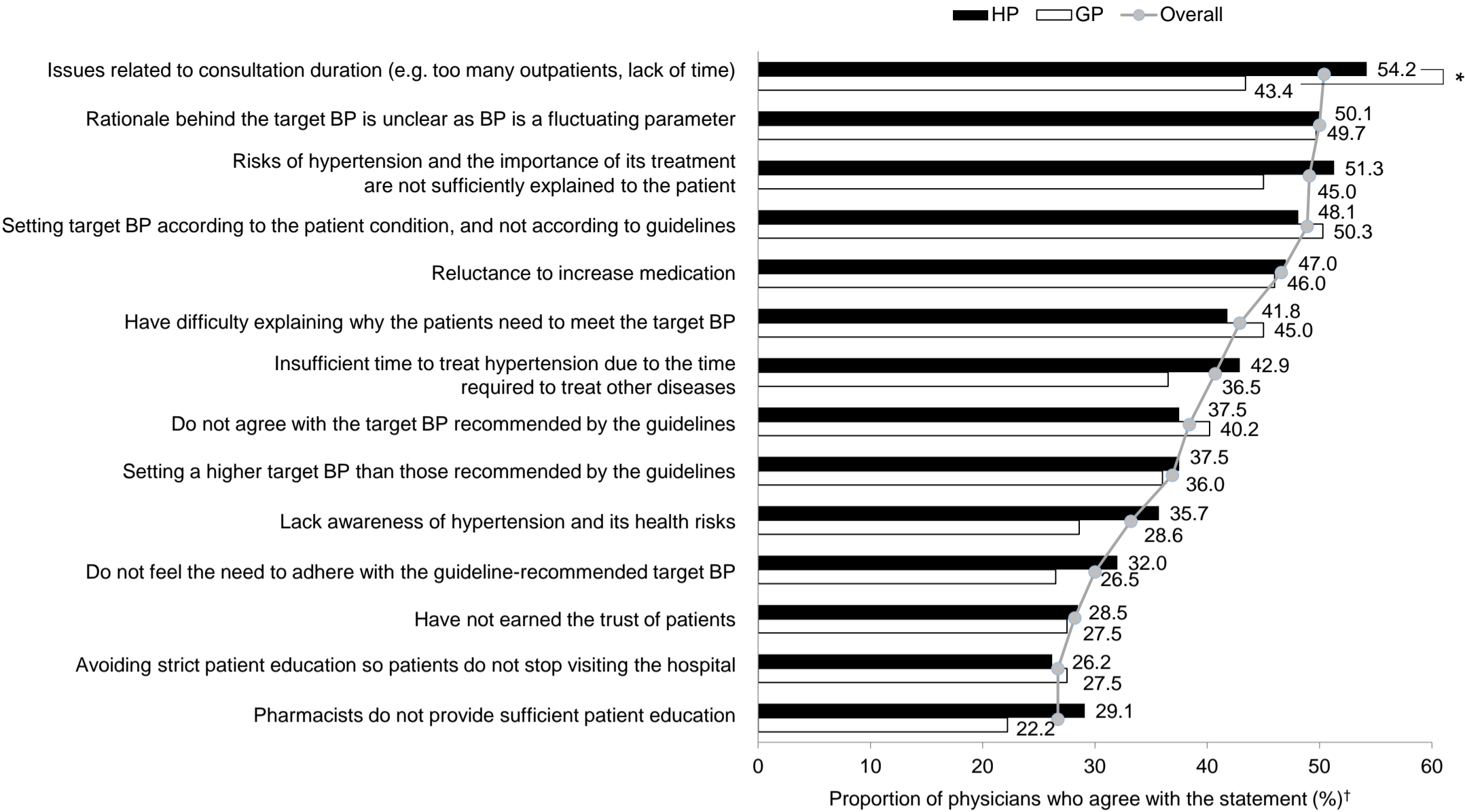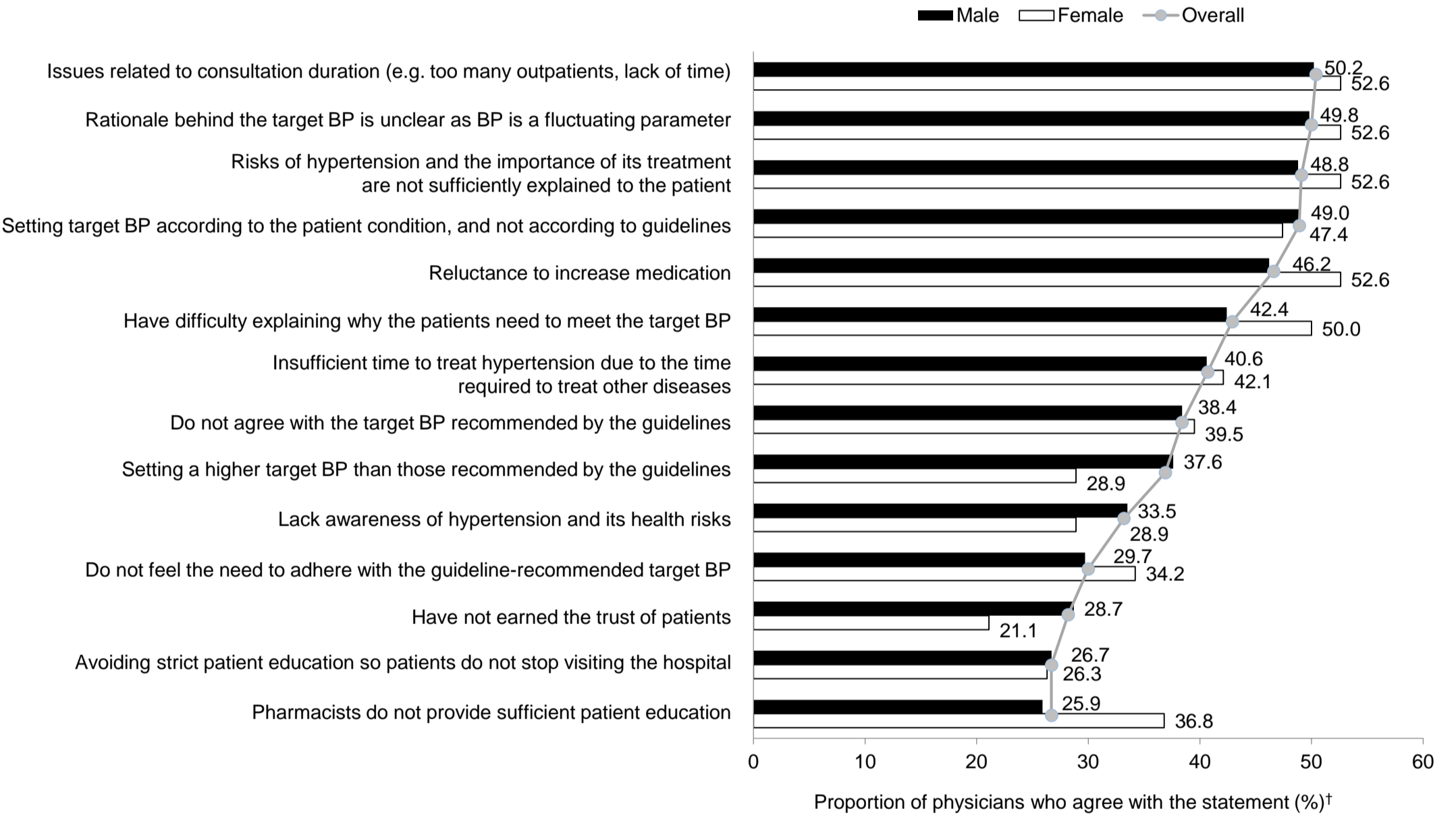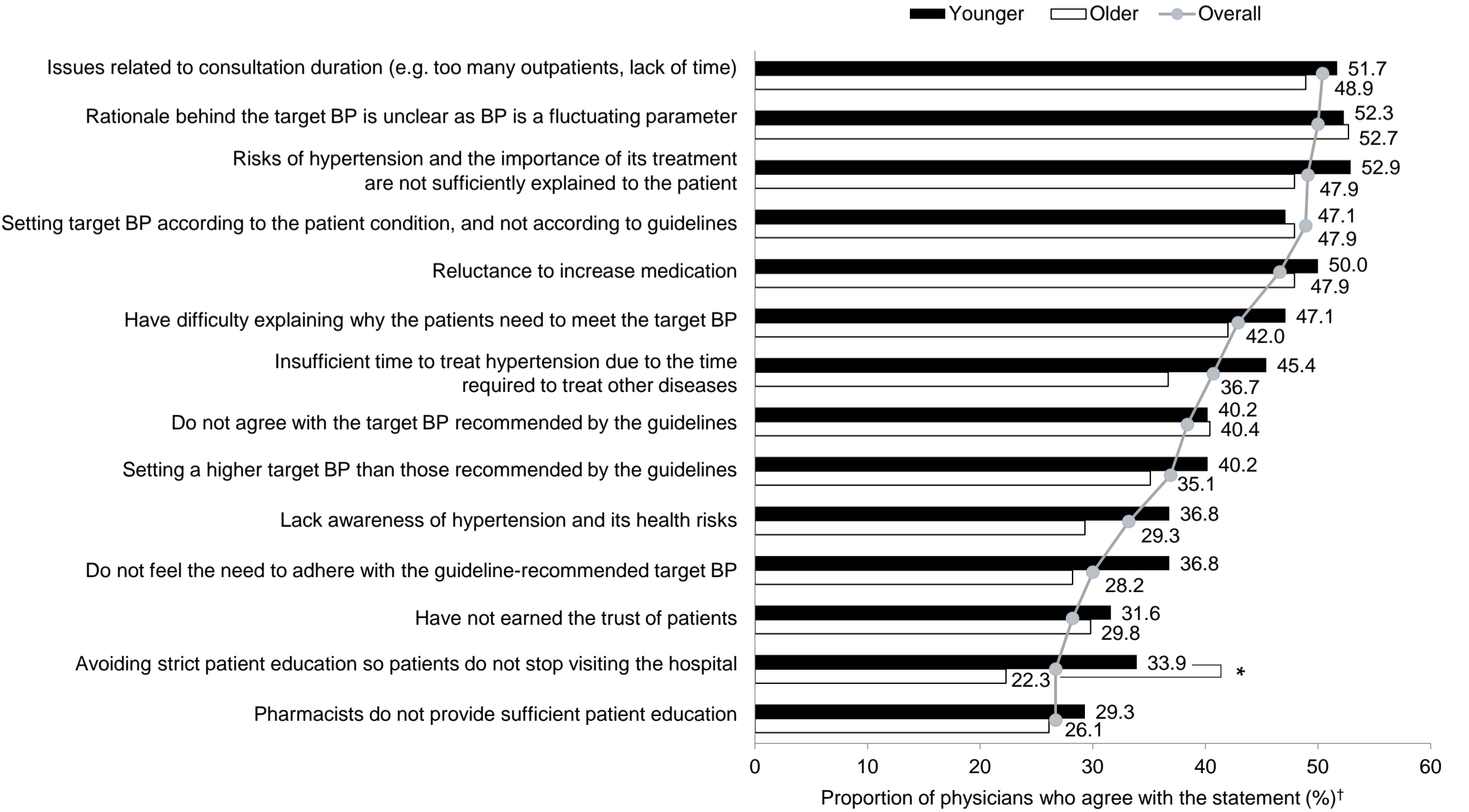

Supplement: Supplementary file 3 — Supplementary Figure 2 [file 41440_2019_365_MOESM3_ESM.pdf]

Supplementary Figure 3

(a)

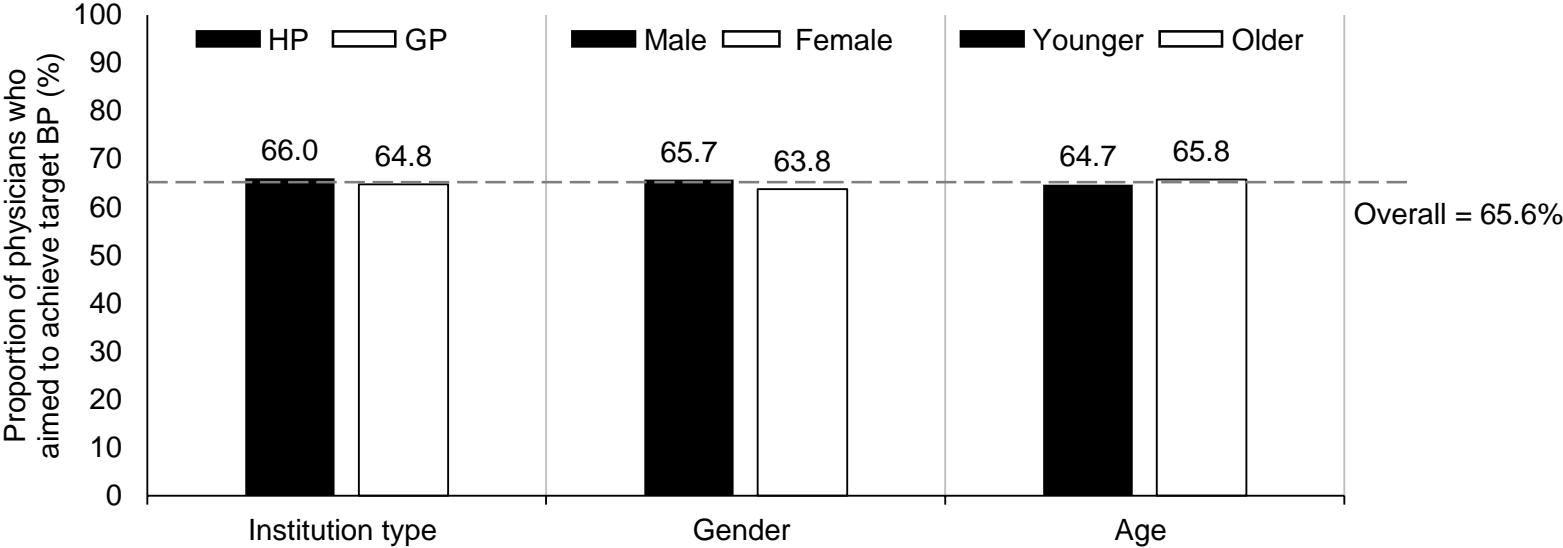

(b)

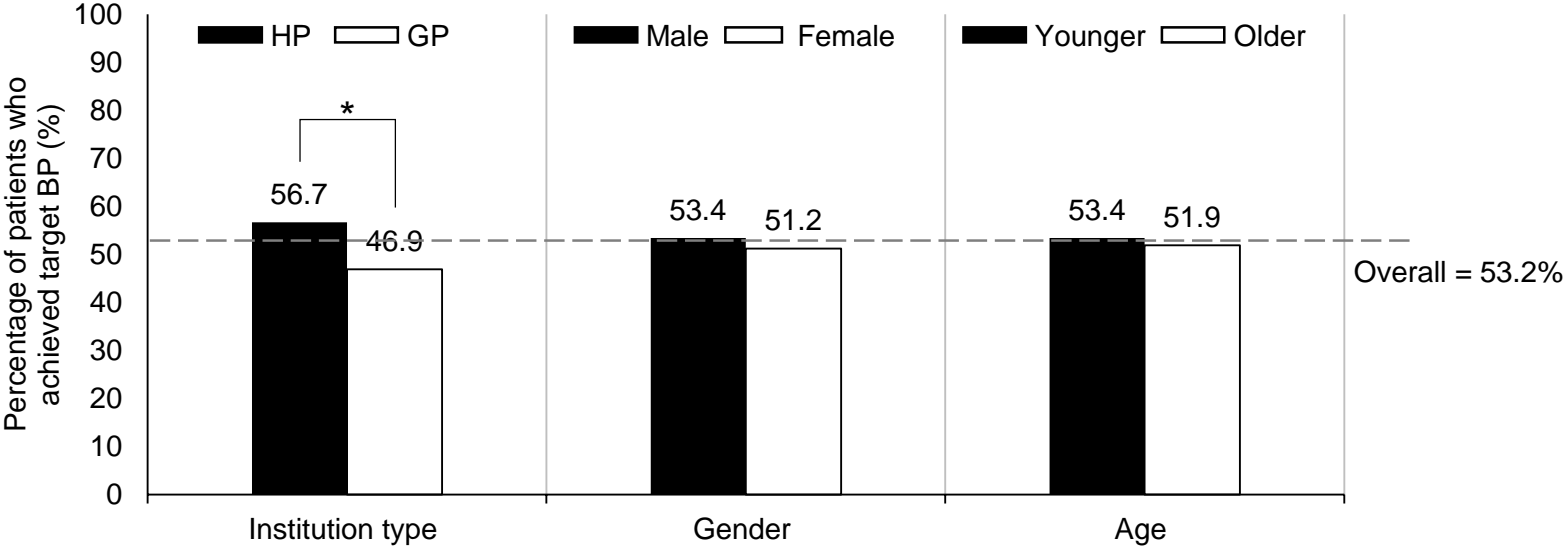

Supplement: Supplementary file 4 — Supplementary Figure 3 [file 41440_2019_365_MOESM4_ESM.pdf]

Supplementary Figure 4

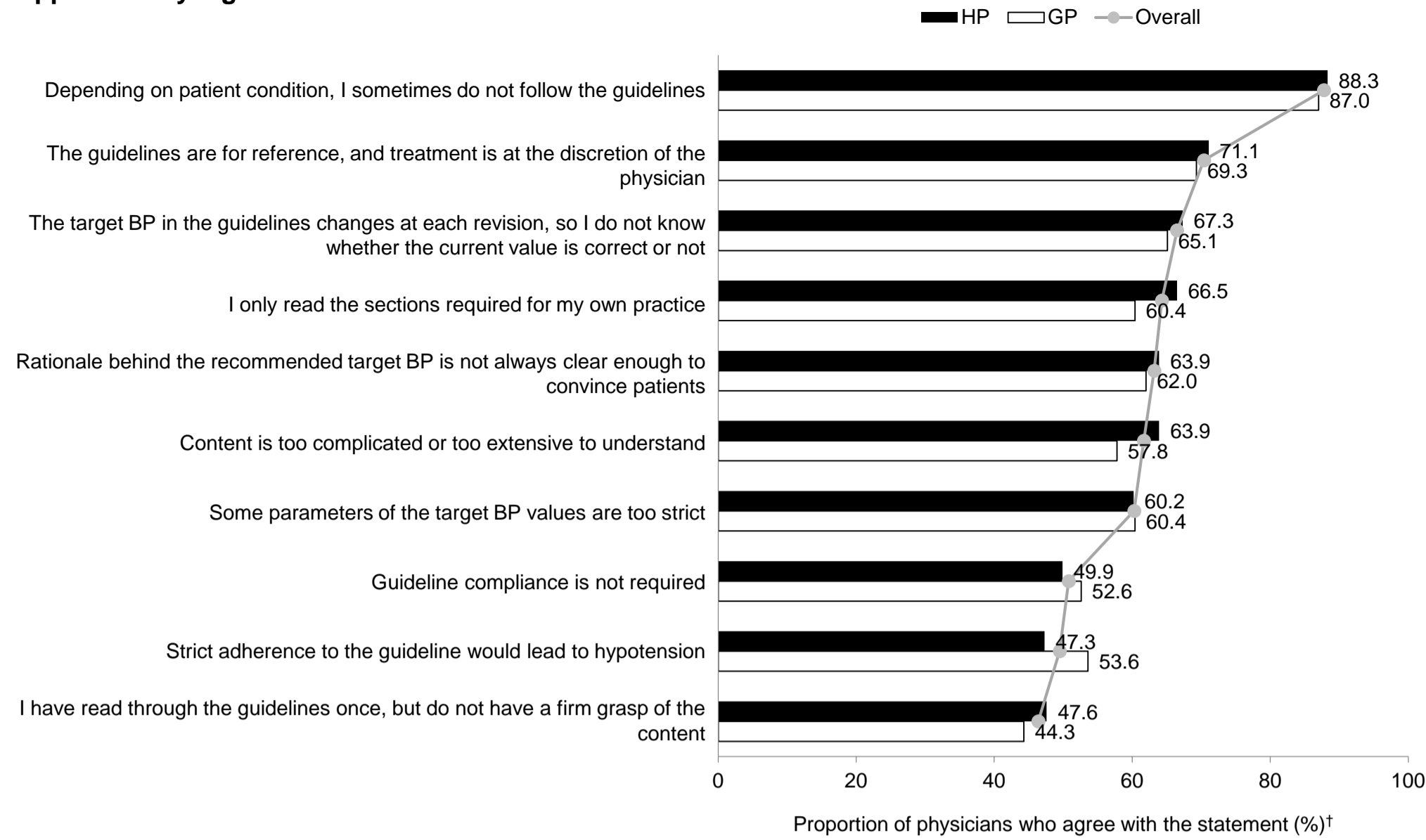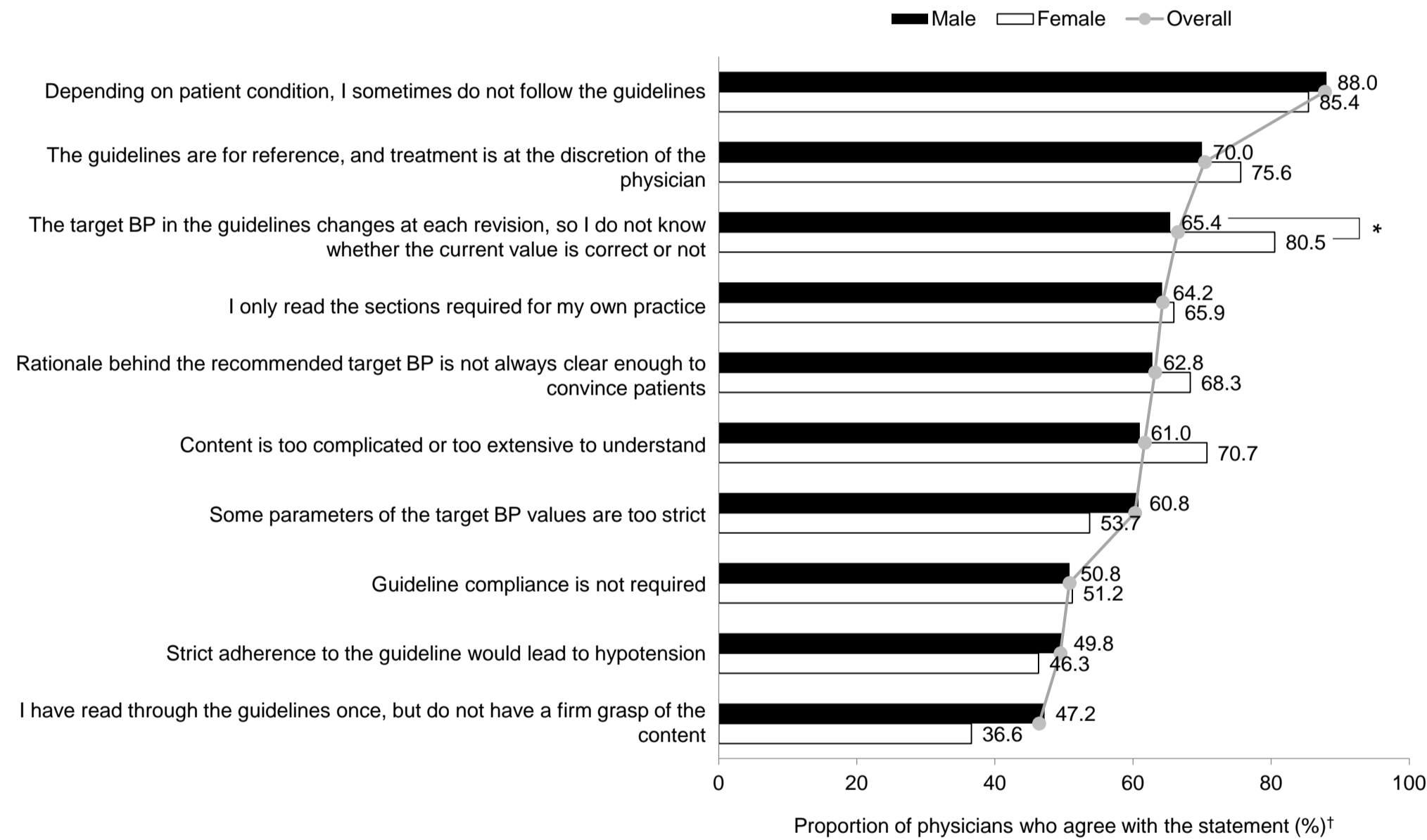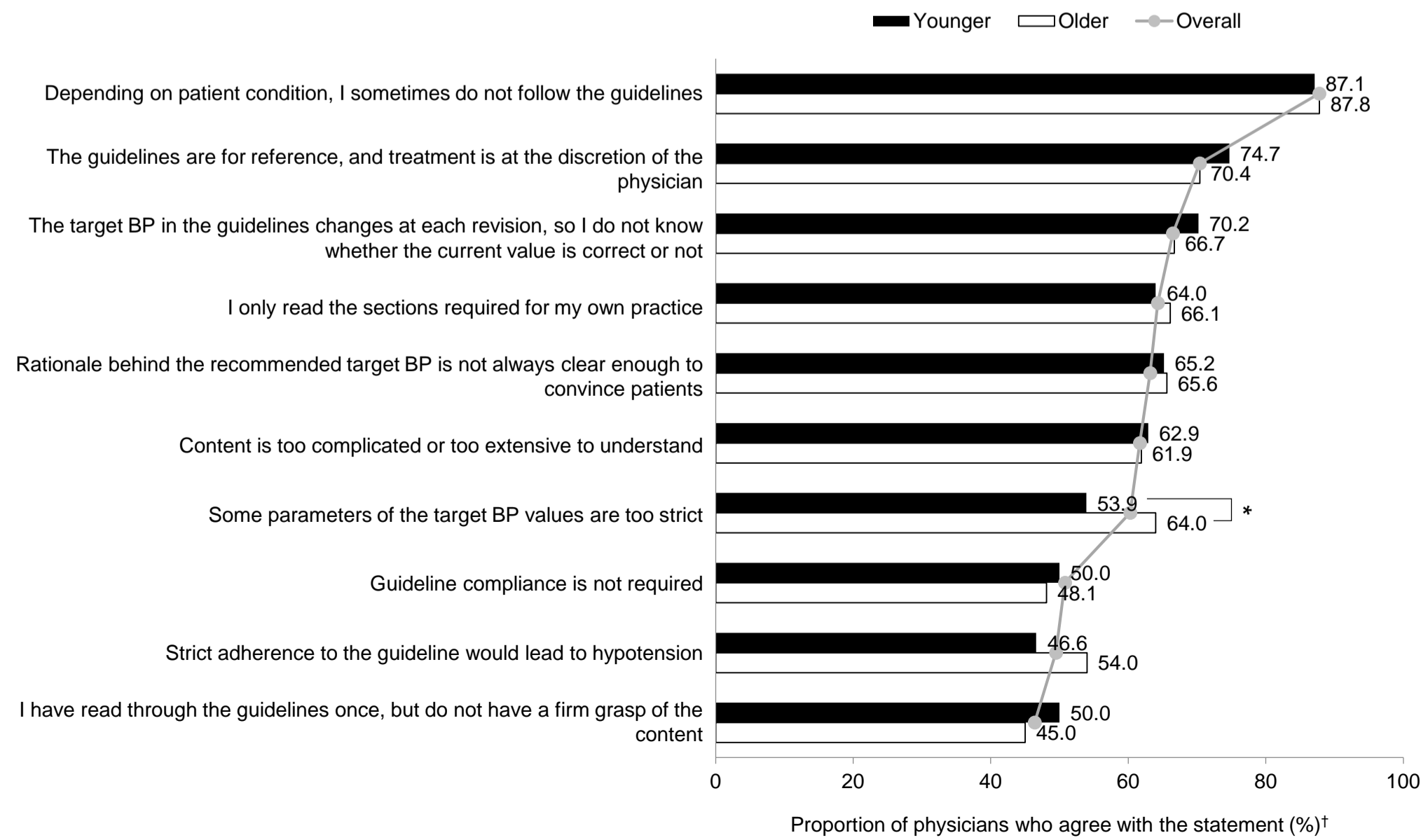

Supplement: Supplementary file 5 — Supplementary Figure 4 [file 41440_2019_365_MOESM5_ESM.pdf]

Supplementary Figure 5

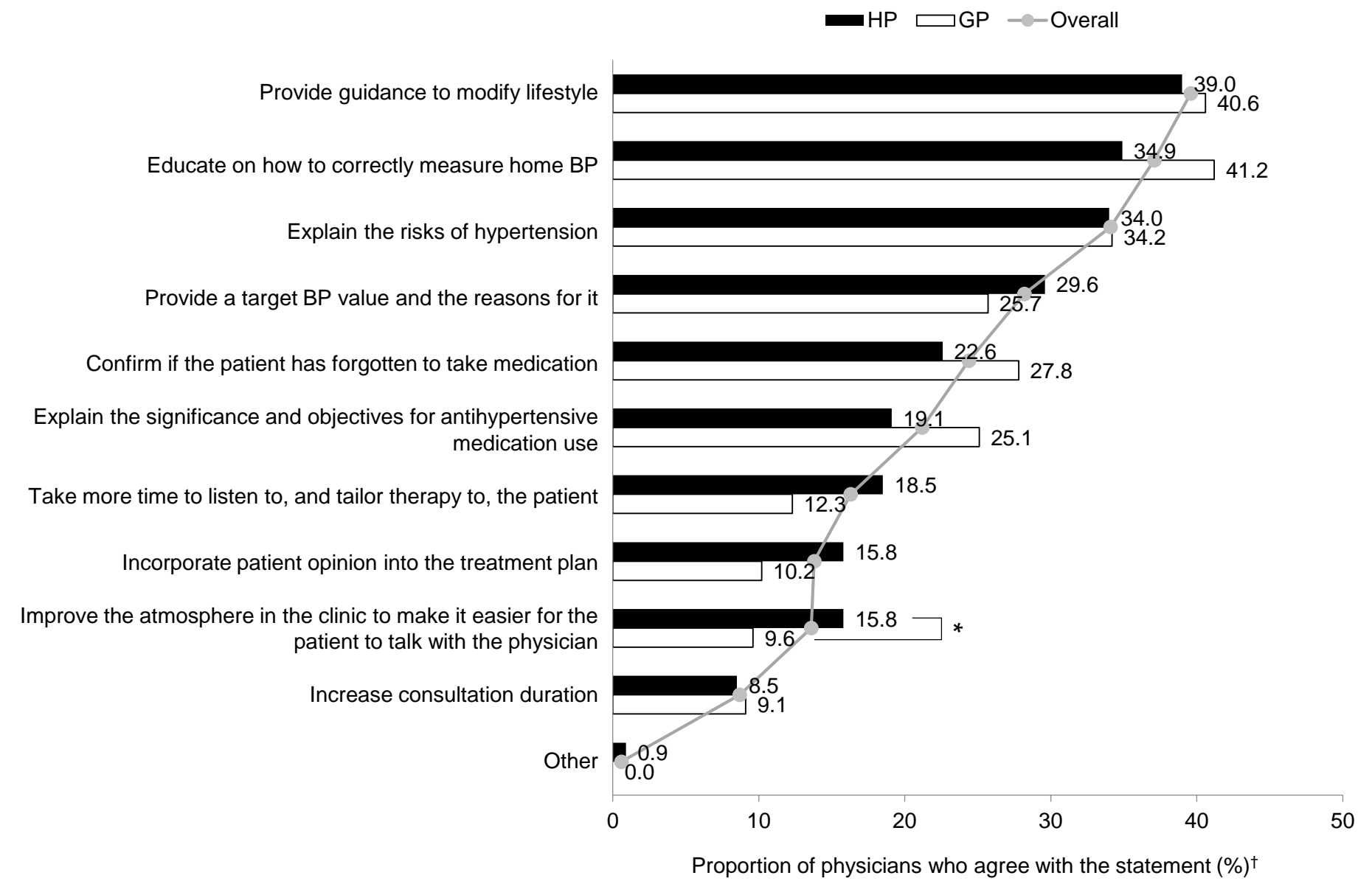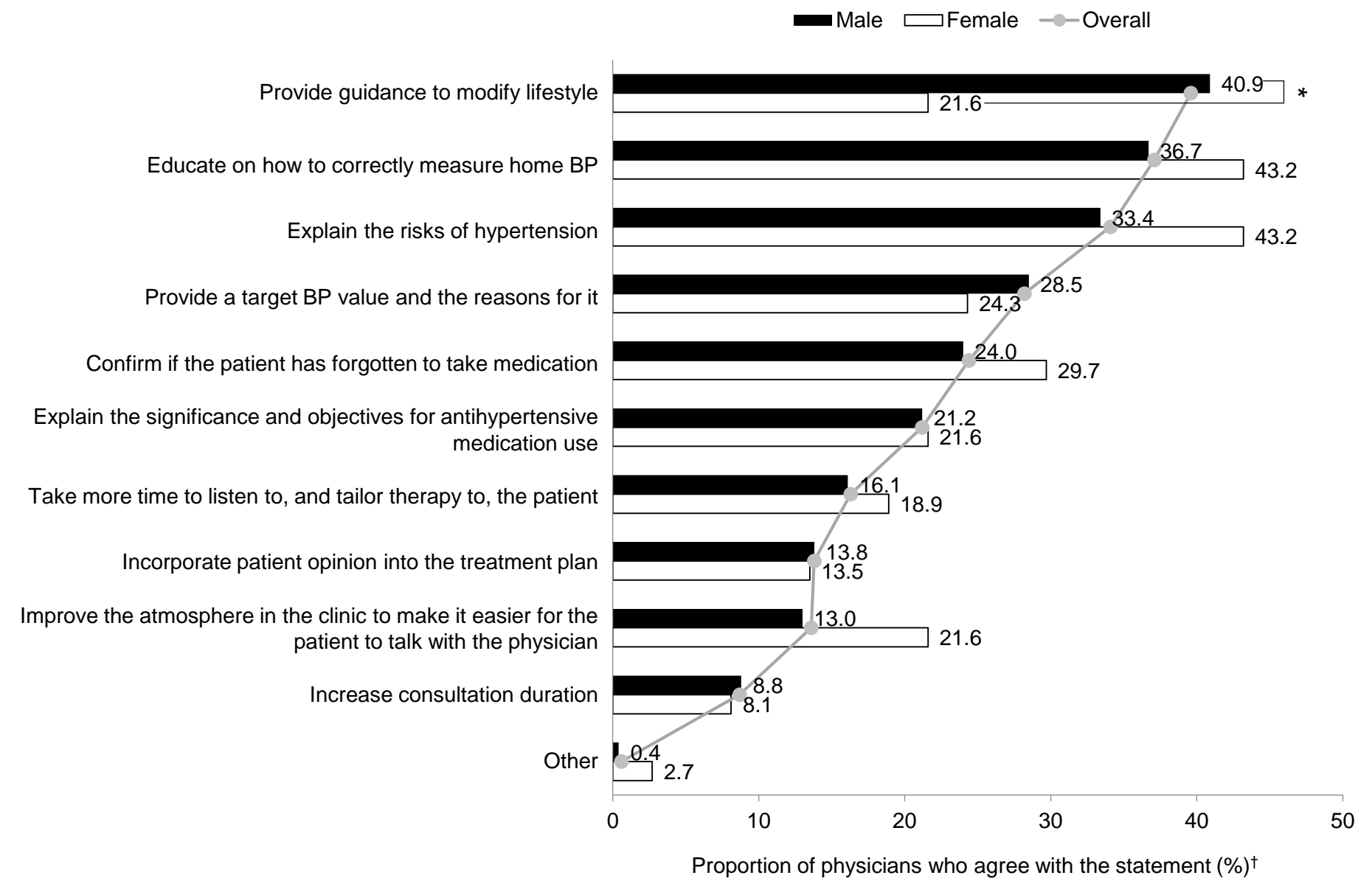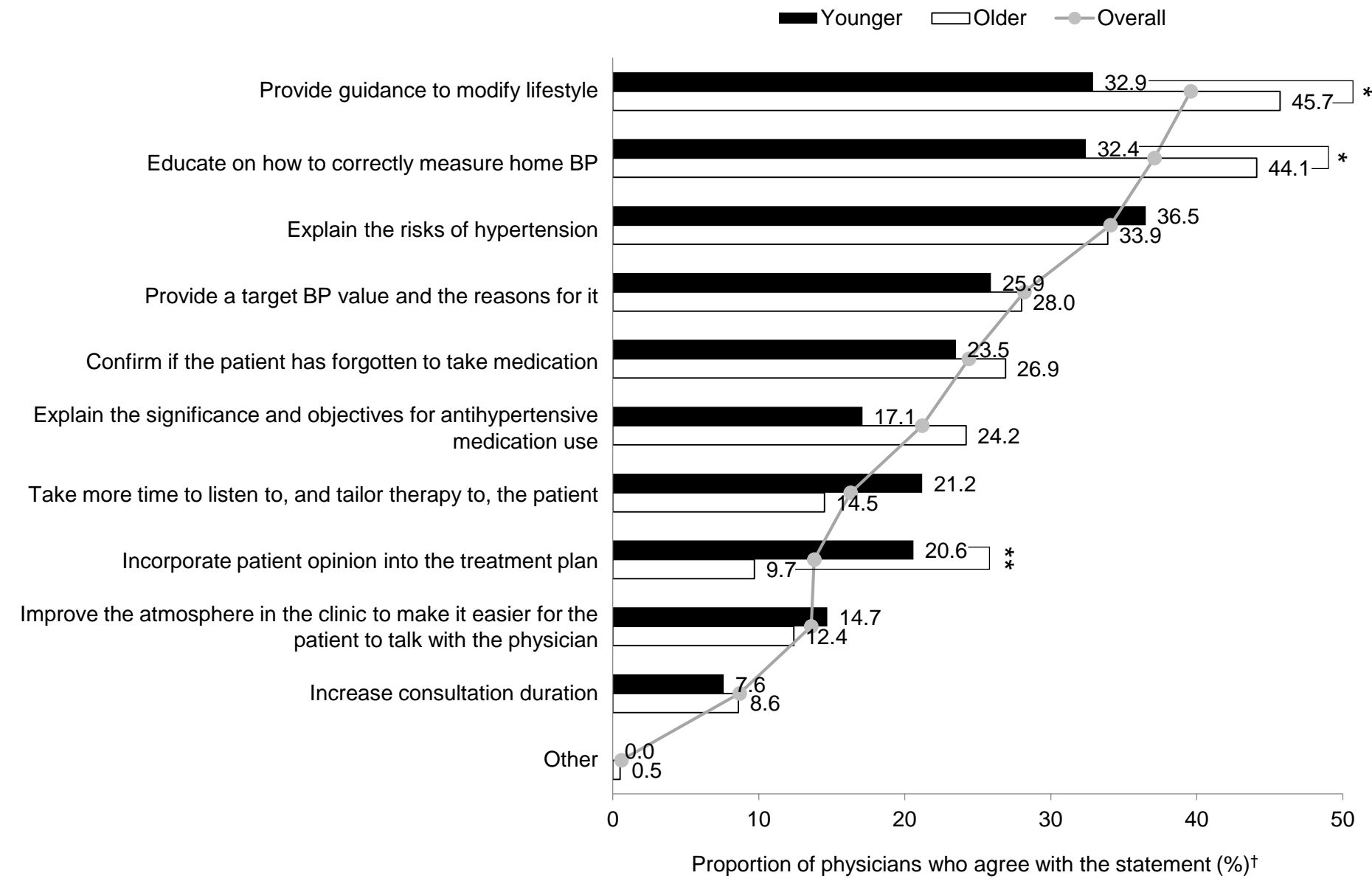

Supplement: Supplementary file 6 — Supplementary Figure 5 [file 41440_2019_365_MOESM6_ESM.pdf]
