# Supplementary material for: Perspectives of patients and physicians regarding hypertensive management from an online survey for excellence: a subanalysis of the PARADOX study by physician categories
Source: Hypertens Res. 2020 Jan 29;43(5):431–41. doi: 10.1038/s41440-019-0365-9 (PMC8075984; doi:10.1038/s41440-019-0365-9)
Supplement: Supplementary file 7 — Supplementary Table 1 [file 41440_2019_365_MOESM7_ESM.docx]

**Supplementary Table 1**. **Physician demographics, by subgroup**

| Parameter | Total | Male | Female | Specialist* | Non- specialist | Hospital physician | General practitioner | Younger age (26–47 years) | | Intermediate age (48–55 years) | Older age (56–95 years) |
| --- | --- | --- | --- | --- | --- | --- | --- | --- | --- | --- | --- |
| Number | 541 | 500 | 41 | 59 | 482 | 349 | 192 | | 178 | 175 | 189 |
| Mean age, years | 51.5 | 52.0 | 46.3 | 49.8 | 51.7 | 49.4 | 55.5 | | 40.7 | 52.0 | 61.3 |
| Male (%) | 92.4 | **100** | **0** | 94.9 | 92.1 | 91.7 | 93.8 | | 86.0 | 93.4 | 97.4 |
| Non-specialist physicians (%) | 89.1 | 88.8 | 92.7 | **0** | **100** | 88.0 | 91.1 | | 84.2 | 92.0 | 91.0 |
| Physicians working in a hospital (%) | 64.5 | 64.8 | 70.8 | 71.1 | 63.7 | **100** | **0** | | 79.8 | 66.1 | 48.7 |
| Mean number of patients prescribed antihypertensives in the last month | 167.1 | 168.5 | 149.9 | 231.8 | 159.2 | 145.5 | 206.4 | | 151.6 | 166.4 | 183.1 |
| Mean time taken for initial/follow-up medical examination (min) | 15.6/ 6.6 | 15.6/ 6.5 | 16.0/ 7.2 | 18.5/ 8.9 | 15.3/ 6.3 | 15.7/ 6.8 | 15.4/ 6.3 | | 15.8/ 6.5 | 15.0/ 6.6 | 16.0/ 6.7 |

*Certified by the Japanese Society of Hypertension.
