# Supplementary material for: Perspectives of patients and physicians regarding hypertensive management from an online survey for excellence: a subanalysis of the PARADOX study by physician categories
Source: Hypertens Res. 2020 Jan 29;43(5):431–41. doi: 10.1038/s41440-019-0365-9 (PMC8075984; doi:10.1038/s41440-019-0365-9)
Supplement: Supplementary file 9 — Supplementary Document 2 [file 41440_2019_365_MOESM9_ESM.docx]

**Supplementary Document 2**. **Physician screening questionnaire**

**Survey on the Management of Hypertension [Physician]**

**Screening Questions**

SC1. Based on your medical records, please tell us the number of patients to whom you prescribed antihypertensive drugs in the last month.

SC2. When prescribing antihypertensive drugs to patients with high blood pressure, do you make decisions on your own?

SC3. Which type of medical institution do you work at? If you work at multiple institutions, please provide an answer for your main place of work.

SC4. Which medical department are you affiliated with? If you are affiliated with multiple departments, please provide the department in which you mainly treat hypertension.

SC5. Are you a specialist doctor certified by the Japanese Society of Hypertension?

SC6. How long was your average consultation with your patient at initial consultation (first visit) and the subsequent visits (follow-up or regular visits)?

SC7. What is your gender?

SC8. What is your current age?

SC9. In which region do you mainly work geographically?
